# Paraoxonase 1 Activity, Polymorphism and Atherosclerosis Risk Factors in Patients Undergoing Coronary Artery Surgery

**DOI:** 10.3390/jcm8040441

**Published:** 2019-03-30

**Authors:** Anna Wysocka, Marek Cybulski, Andrzej P. Wysokiński, Henryk Berbeć, Janusz Stążka, Tomasz Zapolski

**Affiliations:** 1 Internal Medicine in Nursing Department, Medical University of Lublin, 20-090 Lublin, Poland; dojaty@gmail.com; 2 Biochemistry and Molecular Biology Department, Medical University of Lublin, 20-093 Lublin, Poland; marekcybulski@umlub.pl (M.C.); haber100234@gazeta.pl (H.B.); 3 Cardiology Department, Medical University of Lublin, 20-954 Lublin, Poland; a.wysokinski@umlub.pl; 4 Cardiosurgery Department, Medical University of Lublin, 20-954 Lublin, Poland; janusz.stazka@umlub.pl

**Keywords:** paraoxonase, coronary heart disease, risk factor

## Abstract

Background: Paraoxonase1 (PON1), an enzyme connected to high density lipoproteins (HDL) particles, plays an important role in protecting arteries against atherosclerosis. The serum activity and concentration of PON1 depends on several genetic polymorphisms as well as environmental factors. Materials and methods: Investigated population consisted of 71 patients aged 43–76 years with confirmed coronary heart disease (CHD). Established risk factors of CHD such as hypertension, elevated total cholesterol and LDL cholesterol (LDL-C), low HDL cholesterol (HDL-C), diabetes mellitus, obesity, smoking and premature CHD in family history were assessed. PON1 genotype for −108C/T promotor region was determined by polymerase chain reaction-restriction fragments length polymorphism (PCR–RFLP) method. Paraoxonase activity towards paraoxon and arylesterase activity towards phenyl acetate were measured spectrophotometrically. Results: Significant correlations between diabetes mellitus and paraoxonase activity (*R* = −0.264, *p* = 0.026) and between the premature coronary heart disease in family history and PON1 activity (*R* = −0.293, *p* = 0.013) were found. In multivariate analysis, PON1 paraoxonase activity was independently of confounding factors associated with diabetes (OR = 0.985; *p* = 0.024) and premature CHD in family history (OR = 0.983; *p* = 0.027). PON1 activity towards aryl acetate positively correlated with HDL-C level (*R* = 0.255, *p* = 0.032). In patients treated with statins, PON1 paraoxonase activity was significantly (*p* = 0.033) higher than in patients without treatment. Conclusions: In diabetic patients with CHD, paraoxonase activity is lower than in normoglycemic patients despite similar lipid profiles. Diabetes and positive family history in patients with overt CHD are associated with the serum PON1 activity, which might be an additional factor helpful in evaluating cardiovascular risk in this group of patients.

## 1. Introduction

Coronary heart disease (CHD) resulting from atherosclerosis of coronary arteries is the most frequent cause of mortality in highly developed countries. Detailed research to identify the CHD risk factors have been carried out for many years. Age, sex, hypertension, diabetes, obesity, elevated level of total cholesterol and LDL cholesterol (LDL-C), low concentration of HDL cholesterol (HDL-C), smoking, and premature cardiovascular diseases in medical history are regarded as established independent risk factors of CHD [1]. Based on large population studies, it is reported that low concentration of cholesterol transported with HDL fraction is one of the most important risk factors of CHD and high serum HDL concentration plays a protective role [2].

The way in which HDL protect arteries against the damages induced by free radicals and prevent the development of atherosclerotic plaques has been intensively investigated in recent years. An enhanced oxidative stress is regarded as a factor influencing development of atherosclerosis. An important role in antioxidant capacity is attributed to specific enzymes connected to HDL particle such as paraoxonase (PON), glutathione peroxidase (GPX), or platelet activating factor acetyl hydrolase (PAF–AH) [3,4,5]. Paraoxonase1 is one of three enzymes (PON1, PON2, and PON3), coded by a family of genes localized in chromosome 7 [6]. In vitro studies show that this enzyme hydrolyzes peroxidized phospholipids, but the exact mechanisms of PON antioxidant function remain unclear [7,8]. Some studies suggest that decreased activity of PON1 increases the risk of development of atherosclerosis and may be considered as additional strong risk factor for CHD [9,10]. Results of other studies indicate that lower PON1 paraoxonase and arylesterase activities are associated with severity of lesions of coronary arteries in patients with coronary artery disease [11]. High PON1 activity decreases the recurrence of symptoms of CHD and improves prognosis after coronary artery by-pass grafting (CABG) [12]. It was found that PON1 activity is influenced by genetic polymorphism, and studies have been conducted to discover which polymorphic form of PON1 can predict the CHD. It is reported that Q alloenzyme of coding region presents a higher ability to hydrolyze peroxided lipids and more efficiently protects LDL particles against the peroxidation processes than R alloenzyme. Several studies were performed with the aim to prove whether individuals with the isoenzyme PON1 192R are more susceptible for coronary artery disease that persons with the PON1 192Q form. Some experiments confirmed the assumed relationship [13,14], while others obtained the opposite conclusions [15,16]. Some authors reported relationship between the occurrence of allele 55L and atherosclerosis [13,17], whereas other researchers denied this dependence [18,19]. In more recent meta-analysis regarding the association between PON2 311 C/S polymorphism and CHD, authors found a relationship between evaluated polymorphism and CHD occurrence in Caucasians, but not in Asians and Hispanic populations [20]. Wheeler et al. published results of meta-analysis regarding potential relationships between PON1 gene polymorphism in positions −108, 55, 192 and PON2 gene in position 311 of coding region and the risk of CHD. Only a weak association between the occurrence of PON1 allele 192R PON1 and CHD was found [21]. In the prospective Northwick Park Heart Study II, and no correlations between the investigated polymorphisms (PON1 55L/M, 192Q/R, PON2 311C/S, PON3 99A/A) and the occurrence of documented acute cardiovascular incident are reported. However, it was observed that individuals with the genotype PON1 55LM or 55MM, and PON2 311CC were 3.5-fold more susceptible for cardiovascular incident than persons with any other haplotype combination [22]. In a large case-control study assessing possible influence of PON1 status on CHD, no relationship between C-108T and G-909C promoter polymorphism and CHD presence was found. However, the authors noted significantly lower PON1 activity and concentration in patients with CHD in comparison with healthy population regardless of their genotype [23].

Many environmental factors influence paraoxonase activity as well as decreased enzyme concentration and activity was observed independently from the genotype in disorders accelerating the atherosclerosis development such as diabetes, hypercholesterolemia or renal failure. Therefore, it was suggested that evaluation of genotype and enzyme serum activity together may be considered as potential indicators of CHD [24].

The aim of the present study was the evaluation of relationship between PON1 genetic polymorphism, enzyme activity, and other established risk factors of CHD such as hypertension, elevated level of total cholesterol and LDL, low concentration of HDL, smoking, family history of premature CHD and age in patients with confirmed atherosclerosis.

## 2. Materials and Methods

Study population: The study population included 71 unrelated Caucasian individuals (52 men and 19 women), in aged 43–76 years (mean ± SD, 61.09 ± 8.98 years). All patients underwent coronary angiogram that confirmed >50% narrowing of at least one of the major coronary arteries and were admitted to Cardiosurgery Department of Medical University of Lublin for coronary artery by-pass grafting. Classical risk factors of CHD such as age, sex, hypertension, diabetes, overweight/obesity, abnormal lipid profile, smoking and family history of premature cardiovascular disorders as well as previous infarction or statin treatment were evaluated. The levels of total cholesterol and triglycerides greater than 190 mg/dL and 150 mg/dL, respectively, were regarded as hypercholesterolemia and hypertriglyceridemia, respectively. Body mass index (BMI) was calculated as weight in kg divided by height in meters squared and obesity was recognized if BMI was above 30 kg/m^2^ and overweight when BMI was above 25 kg/m^2^. Hypertension was defined as diastolic blood pressure greater or equal to 140/90 mmHg and/or hypertension treatment and diabetes as a fasting glucose level greater or equal 126 mg/dL and/or treatment with hypoglycemic drugs. Smoking was regarded as everyday smoking of at least one cigarette. Positive family history was defined as the occurrence of cardiovascular system diseases in at least one of first-degree relatives younger than 60 years.

The study protocol was approved by the local ethics committee (decision of Bioethics Committee of Medical University of Lublin No KE-0254/76/2002). Written informed consent was obtained from all of the participants. The investigation was performed according to the principles outlined in the Declaration of Helsinki.

Blood samples for analysis of lipid profile, PON1 activity and polymorphism were collected before the surgery, through venipuncture in heparin or EDTA coated tubes.

### 2.1. DNA Extraction and Analysis

Genomic DNA was extracted from the venous blood using the Gen Elute™ Blood Genomic DNA kit (Sigma) according to manufacturer’s instructions. PON1 genotype for −108 was determined by PCR–RFLP method. Briefly, the primers previously described by Brophy et al. [25] were used to amplify −108C/T promotor polymorphism region. DNA (1 μg) was denatured at 94 °C for 4 min (“hot start”) and then amplified for 40 cycles: each cycle comprised denaturation at 94 °C for 50 s, annealing at 64 °C for 30 s, and extension at 72 °C for 30 s, with final extension time of 7 min. The PCR product (119 bp) was digested with Bsh126I (Fermentas) at 37 °C for 12 h. The digested products were separated by 2% agarose gel electrophoresis and identified by ethidium bromide staining in UV light. Allele C corresponds to the presence of a non-digested 119 bp fragment, while allele T corresponds to 2 digestion fragments of 52 and 67 bp.

### 2.2. Paraoxonase Activity

Paraoxonase and arylesterase activities were determined according to Eckerson et al. [26]. PON1 activity towards paraoxon was evaluated by measuring absorption at 412 nm using continuously recording spectrophotometer (DU 640; Beckman) after introducing serum to 50 mM glycine/NaOH buffer (pH 10.5) containing 1.0 mMparaoxon, and 1.0 mM CaCl_2_. Enzyme activity was calculated with a molar extinction coefficient of 18,290 M^−1^ cm^−1^. One unit of paraoxonase activity produced 1 nmol of p-nitrophenol per minute. PON1 activity towards phenyl acetate was measured in 20 mM TrisCl buffer (pH 8.0) containing 1 mM substrate and 1 mM CaCl_2_. The absorbance was monitored spectrophotometrically at 270 nm. Enzyme activity was calculated with a molar extinction coefficient of 1310 M^−1^ cm^−1^. One unit of arylesterase activity hydrolyzed 1 μmol of phenyl acetate per minute.

### 2.3. Lipid Profile

Concentrations of total cholesterol, triglycerides and HDL-C were evaluated by specific enzymatic techniques. LDL-C level was calculated by the Friedewald formula. Cardiac index was calculated as total cholesterol/HDL-C ratio.

### 2.4. Statistical Analysis

Data were statistically analyzed using the Statistica ver. 13 (Dell Inc., Tulsa, OK, USA) software. Continuous variables were compared using the non-parametric Mann–Whitney test. Discontinuous variables were estimated by the χ^2^ test. Univariate correlations were calculated using Spearman test. Univariate analysis was followed by multiple logistic regression analysis to estimate independence of correlations noted in the univariate test. Logistic regression analysis was used to determine the odds ratios (OR) with 95% confidence intervals (CI) between diabetes and positive family history and independent covariates. *p* values less than 0.05 were considered significant. Data in plots are presented as median, 25–75 percentiles, and min–max values.

## 3. Results

**Figure 1:**
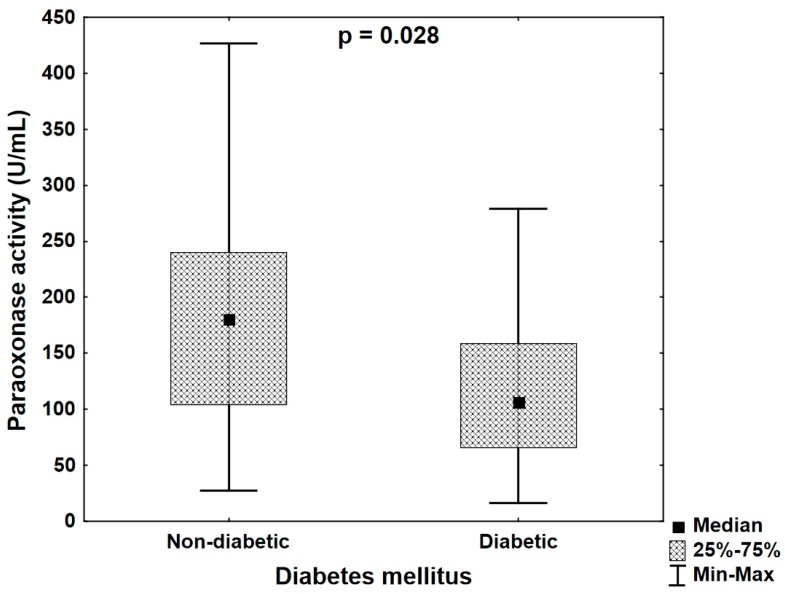
Paraoxonase activity in the groups of non-diabetic and diabetic patients (Mann–Whitney U test).

We evaluated the relationships between paraoxonase and arylesterase activities of PON1, and classic risk factors of CHD, including sex, age, hypertension, diabetes mellitus, obesity or overweight, cigarette smoking and medical history of CHD as well as previous infarct and statin treatment. Clinical and biochemical characteristics of the subjects investigated in our study are summarized in the Table 1. Additionally, associations of PON1 gene polymorphism, PON1 activity and serum lipid profile parameters as total cholesterol concentration, level of LDL cholesterol, level of HDL cholesterol, triglycerides concentration and total cholesterol/HDL cholesterol ratio (cardiac index) were assessed. The correlations between serum PON 1 activity, and risk factors of CHD in the group of patients is presented Table 2. We discovered a significant negative correlation between diabetes mellitus and PON1 paraoxonase activity (*R* = −0.264, *p* = 0.026). The median paraoxonase activity in the group of patients with diabetes 106.69 (16.40–278.84) U/mL was significantly lower (*p* = 0.028) than its value 180.43 (27.34–426.46) U/mL in the group of non–diabetic patients (Figure 1). Among patients with CHD, TT genotype occurred more frequently in diabetic patients than in patients without the diabetes but the difference did not attain a significant level (*p* = 0.348).

**Figure 2:**
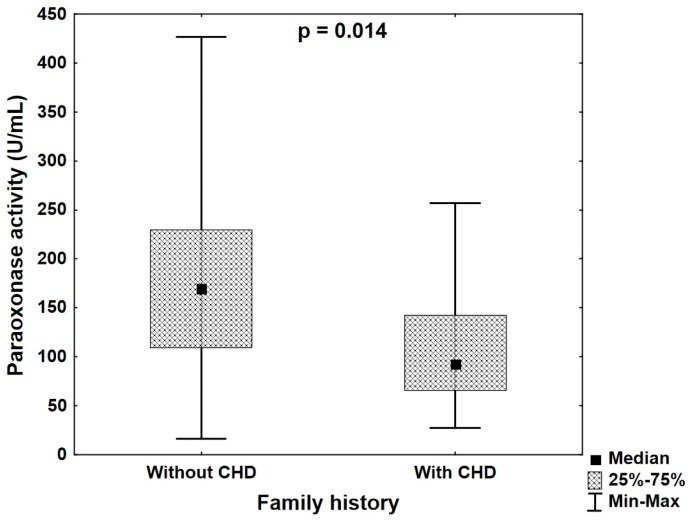
Paraoxonase activity in the groups of patients without coronary heart disease (CHD) in family history and with coronary heart disease in family history (Mann–Whitney U test).

The correlation analysis revealed the significant correlation between the premature CHD in family history and PON1 paraoxonase activity (*R* = −0.293, *p* = 0.013). PON1 paraoxonase activity in the group of patients with CHD in family history ranged from 27.34 to 256.97 (median 92.95) U/mL and was significantly lower (*p* = 0.014) than in patients without positive family history (range 16.40–426.46; median 169.49 U/mL) (Figure 2).

In obese and overweight patients, only the trend (*p* = 0.078) towards lower values of median PON1 paraoxonase activity 158.56 (27.34–393.66) U/mL in comparison with normal-weight patients 224.17 (16.40–426.46) U/mL was observed. In normal weight patients if compared with obese and overweight patients, the tendency to more frequent occurrence of CC genotype (*p* = 0.07) was found.

**Figure 3:**
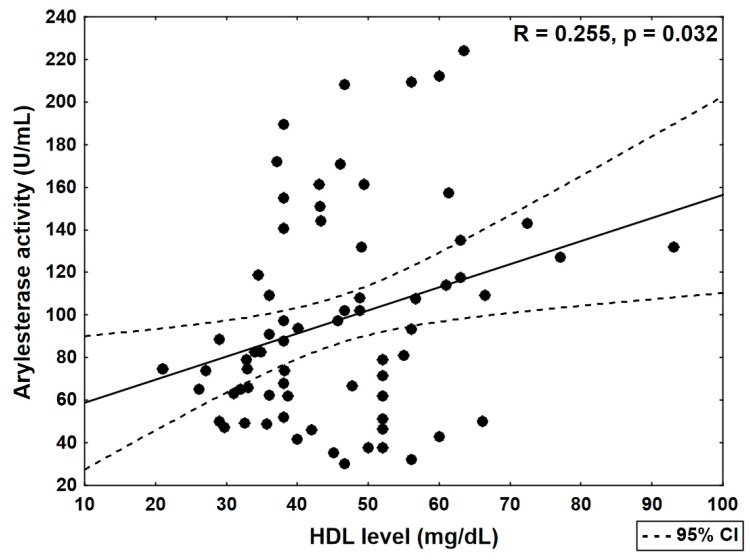
Correlation of HDL concentration and arylesterase activity in the group of patients with coronary heart disease (Spearman’s rank test). CI, Confidence Interval.

HDL concentration was significantly correlated with PON1 arylesterase activity (*R* = 0.255, *p* = 0.032; Table 3 and Figure 3). In women, significantly higher (*p* = 0.02) HDL concentration in comparison with men was noted. No significant relationships between the PON1 activity and the age of patients and tobacco smoking were observed.

Comparing subgroups of patients treated and not treated with statins, we found a significant (*p* = 0.033) difference in PON1 activity against paraoxon (Table 4). Levels of total cholesterol and LDL-C were lower in patients without statins treatment, but the difference did not attain statistical significance. Only the trend (*p* = 0.069) regarding LDL-C was observed. Serum concentration of HDL-C (*p* = 0.398) and cardiac index (*p* = 0.137) did not differ significantly between analyzed subgroups. In patients treated with statins, the level of triglycerides was significantly higher (*p* = 0.008) in comparison with patients without statin treatment. Comparing particular statins (atorvastatin, simvastatin, lovastatin and fluvastatin) to each other, we did not find any significant differences in lipid profile and PON1 activity in investigated subgroups of patients being treated with these drugs independently from the dose. 

Multiple logistic regression was used to evaluate whether PON1 activity was associated with outcomes (family history of cardiac episode or diabetes mellitus) independently of other confounders. We confirmed significant negative association of PON1 paraoxonase activity with diabetes (*p* = 0.024, Table 5) and positive family history (*p* = 0.027, Table 6) and these associations were independent of sex, levels of HDL-C, LDL-C triglycerides, previous infarction and genotype, which were introduced to the regression models as covariates. Total cholesterol level was not included in the regression model because it was strongly correlated with LDL (*r* = 0.847).

## 4. Discussion

In our study, we found significant correlation of PON1 arylesterase activity and HDL-C level and significantly lower PON1 paraoxonase activity in patients with diabetes and positive family history. PON1 paraoxonase activity was associated with diabetes and positive medical history of CHD independently of other factors. Results of our study are in overall agreement with previously reported research regarding association of PON1 activity with atherosclerosis risk factors, although we show some valuable findings.

Because of the physical connection of PON1 with the HDL particle (only 5% of enzyme activity is connected with VLDL and chylomicrones) [27], the correlation of PON1 activity and HDL serum concentration was primarily assumed. Our study showed significant correlation between PON1 arylesterase, but not paraoxonase activity, and HDL level. Similar results were obtained by authors evaluating healthy population, who discovered significant positive correlation of PON1 activity and HDL concentration [28] as well as interactions between high TG, high LDL-C and low HDL-C level and lower PON1 activity [29]. In contrast, investigators evaluating patients with CHD did not found any relationships between PON activity and total cholesterol HDL-C, LDL-C or TG concentrations [30,31]. Participants of our study were characterized by median HDL-C of 45.48 ± 13.62, which may be considered as normal [1]. According to authors of the above-mentioned studies, PON1 activity depends on the number of enzyme particles connected to certain HDL molecule rather than on total HDL serum concentration, which is why normal, comparable with healthy population, concentrations of HDL do not exclude lower PON1 activity, which is more typical for CHD.

Undoubtedly, it should be remembered that altered PON1 activity and serum lipid profile in patients with CHD may result from their treatment with drugs lowering the cholesterol level, which modify the serum lipid profile and PON1 activity itself. In our study, almost 70% of patients were treated with statins, but differences of total cholesterol, LDL-C, HDL-C levels and cardiac index, a valuable index for risk of atherosclerosis, between subgroups with and without treatment were not significant. Moreover, in the whole investigated group, mean values of HDL-C and cardiac index were normal. Despite similar levels of all lipid profile compounds, which are potentially affected by statins in both analyzed subgroups, we found significantly higher PON1 paraoxonase activity in patients treated with statins. This finding may indicate that PON1 activity in patients with atherosclerosis may be considered as an additional marker useful for monitoring of treatment with statins. Many studies evaluating how different statins influence PON1 activity are provided [32,33,34], a recently published meta-analysis [35] confirms, independent of particular drug, dose, treatment duration and serum lipid profile, an effect of enhancing PON1 activities and concentration. Additionally, it is described that individual response to treatment with statins is dependent on PON1 Q192R polymorphism [36], which may explain the significant difference of PON1 paraoxonase activity between patients with and without treatment noted in our study.

A potentially important finding of our study is demonstrating significantly lower PON1 serum activity in patients with CHD and diabetes mellitus in comparison with normoglycemic patients with CHD. Additionally, we found that PON1 paraoxonase activity is independently associated with diabetes. Currently, a lot of attention is paid to possible relationships of PON1 activity and diabetes, but results are not univocal. Our study confirmed data showing decreased PON1 activity in patients with diabetes mellitus [37]. This finding may be explained by multiple mechanisms. Firstly, the glycation process of proteins (also PON1 molecule) that is characteristic for diabetes impairs their physiological functions, which may lead to decrease in PON1 activity and the serum antioxidative efficiency [38]. Moreover, glycated proteins more easily undergo catabolic processes and synthesis of proteins in diabetes is generally decreased [39]. Secondly, as a result of the oxidative–antioxidative imbalance, more oxidized LDL particles are produced, which are natural substrates for PON1—in vivo more particles of enzyme may be used up in defense against peroxidation. Additionally, PON1 particles undergo peroxidation, which provides an impairment of their enzymatic function. We noted association of diabetes and PON1 paraoxonase activity, which is considered as highly dependent on genetic factors. Previously, it was proved that PON1 concentration and activity are affected almost exclusively by −108C/T polymorphism [40]. We found no significant difference between diabetic and non-diabetic carriers of C/T alleles, but, in the presence of the allele 192RR in the PON1 gene coding region enzyme, PON1 activity towards paraoxon was described as higher than in the presence of alloenzyme QQ [24]. In a recently performed meta-analysis, it was proved that in European population allele R represents protective factor in development of diabetes [41]. We may suppose that frequency of R allele was lower in our group of patients with diabetes than in non-diabetic participants, hence we recorded lower paraoxonase activity in diabetics. Additionally, it may be supposed that, because *R* isoenzyme (preferably hydrolyzing artificial substrate paraoxon) is more sensitive to inactivation by oxidative stress and inflammation [42], PON1 paraoxonase activity is more strongly affected than arylesterase activity. Comparing subgroups of diabetic and non-diabetic patients, we did not find significant difference in HDL-C level. The decreased PON1 serum activity observed in diabetic patients despite similar non-diabetics lipid profile, may result from the notified tendency of connecting the PON1 rather to bigger HDL_2_ particles [43], while in the serum of diabetics lower density lipoproteins easily undergoing the peroxidation process and HDL_3_ are dominant fractions. Recently, an association between PON1 activity and type 2 diabetes mellitus regardless of total cholesterol, large, medium and small particles of LDL, HDL, IDL and VLDL cholesterol concentrations is presented [44], but the authors did not evaluate genetic polymorphism and CHD occurrence in patients was not investigated. It is known that in patients with diabetes the risk of atherosclerosis development and its complications is higher than in normoglycemic patients [45]. The assessment of only classical lipid profile may be insufficient and erroneous for evaluating individual cardiovascular risk in this group of patients. In view of these considerations, there is the clinical rationale that PON1 activity may be additional valuable factor helpful in evaluating cardiovascular risk in diabetic patient.

In our study, only a trend to lower PON1 serum activity was observed in obese or overweight patients. Considering the reported negative correlation of PON1 serum activity and BMI in extremely obese patients [46] and association of PON1 activity with prolonged hyperleptinemia dependent on adipose tissue amount [47], we may assume that median BMI in our investigated group was insufficient to affect significantly PON1 activity.

The found association of medical history of premature cardiovascular disease and PON1 paraoxonase activity may emphasize the importance of genetic factors underlying CHD, because, as discussed above, PON1 activity towards paraoxon is highly variable depending on genetic polymorphism. However, no significant differences in genotypes frequency between patients with or without family history of cardiovascular incidents were observed, as only the C/T polymorphism of PON1 gene promotor region was evaluated. The explanation of this fact may be high genetic variability as well as linkage disequilibrium between different alleles in coding and promoter region, which influences PON1 activity towards different substrates [48]. Additionally, greatly variable relationship between different genotypes and particular fractions of lipoproteins levels should be considered [49].

We are aware of some limitations regarding presented study, primarily the few examined patients with confirmed CHD as well as the limited number of investigated polymorphisms. We hope that our study allows underlining the importance of investigations of additional, not only classical, risk factors of CHD and a need to provide prospective studies including larger groups of patients.

Results of our study may lead to the conclusion that investigating only individual compounds of lipid profile in patients with overt severe atherosclerosis may cause an underestimation of cardiovascular risk. An assessment of the plot of components consisting of lipid profile, antioxidant-enzyme activity against different substrates and genetic factors allows for more complete evaluation of patient status. PON1 activity might be an additional valuable marker of overall cardiovascular risk and helpful in providing individual, personally tailored treatment with lipid lowering drugs.

## 5. Conclusions

1. In diabetic patients with CHD, paraoxonase activity is lower than in normoglycemic patients despite similar lipid profiles.
2. Diabetes and positive family history in patients with overt CHD are associated with the serum PON1 activity, which might be an additional factor helpful in evaluating cardiovascular risk in this group of patients.

## Figures and Tables

**Table 1:** Baseline characteristic of investigated patients.

	Group of Patients (*n* = 71)
Men (*n*/%)	52/73.24
Women (*n*/%)	19/26.76
Age (years ± SD)	61.09 ± 8.98
Total cholesterol level (mg/dL ± SD)	197.86 ± 47.29
LDL cholesterol (mg/dL ± SD)	122.02 ± 39.43
HDL cholesterol (mg/dL ± SD)	45.48 ± 13.62
Triglycerides (mg/dL ± SD)	155.95 ± 107.18
Body mass index (±SD)	27.96 ± 4.39
Smoking (*n*/%)	19/26.76
Hypertension (*n*/%)	46/64.79
Previous myocardial infarct (*n*/%)	36/50.70
Coronary heart disease in family history (*n*/%)	10/14.08
Diabetes (*n*/%)	14/19.72
Lipid lowering drugs	51/71.08
- statins	49/69.01
- fibrates	2/2.82

**Table 2:** Correlation of serum paraoxonase activity and risk factors of coronary heart disease in group of patients (*R*, correlation coefficient; *p*, value according to Spearman test).

	*R*/*p*	Age	Diabetes Mellitus	Smoking	Family History	BMI
Paraoxonase activity (U/mL)	*R*	0.020	−0.264	−0.174	−0.293	−0.100
	*p*	0.875	0.026	0.147	0.013	0.416
Arylesterase activity (U/mL)	*R*	0.087	0.150	−0.144	−0.020	0.125
	*p*	0.484	0.211	0.230	0.870	0.300

**Table 3:** Correlation of PON1 activity towards paraoxon and phenyl acetate and serum lipid profile in group of patients (*R*, correlation coefficient; *p*, value according to Spearman test).

	*R*/*p*	Total Cholesterol Level (mg/dL)	HDL Level (mg/dL)	LDL Level (mg/dL)	Triglycerides Level (mg/dL)	Total Cholesterol/HDL Index
Paraoxonase activity (U/mL)	*R*	−0.130	−0.054	−0.130	0.110	−0.042
	*p*	0.281	0.657	0.281	0.361	0.728
Arylesterase activity (U/mL)	*R*	0.171	0.255	0.161	−0.016	−0.118
	*p*	0.153	0.032	0.180	0.895	0.328

**Table 4:** Comparison of lipid profile and PON1 activity towards paraoxon and phenyl acetate in subgroups of patients treated or not treated with statins (Mann–Whitney U test).

	Patients Treated with Statins (*n* = 49)	Patients Untreated with Statins (*n* = 22)	*p*
Total cholesterol (mg/dL ± SD)	195,265 ± 49,211	201,227 ± 41,300	0.546
HDL (mg/dL ± SD)	46,133 ± 13,393	43,745 ± 13,788	0.398
LDL (mg/dL)	115,461 ± 35,270	135,518 ± 43,545	0.069
Triglycerides (mg/dL ± SD)	174,071 ± 117,520	109,818 ± 48,735	0.008
Total cholesterol/HDL index ± SD	4525 ± 1646	5036 ± 1924	0.317
PON1 paraoxonase activity (U/mL ± SD)	183,980 ± 90,846	134,443 ± 76,330	0.033
PON1 arylesterase activity (U/mL ± SD)	99,026 ± 54,013	93,122 ± 34,562	0.917

**Table 5:** Results of logistic analysis for association of diabetes mellitus with independent covariates.

	*p*	Odds Ratio
HDL-C level	0.825	0.993
LDL-C level	0.058	0.978
TG level	0.884	1.000
PON1 paraoxonase activity	0.024	0.985
PON1 arylesterase activity	0.348	1.008
Family history of cardiac incidents	0.988	1.210
CC + CT genotype	0.887	1.246
TT genotype	0.904	1.112
Previous myocardial infarction	0.031	5.314
Sex	0.871	0.850

**Table 6:** Results of logistic regression analysis for association of family history of cardiac episode with independent covariates.

	*p*	Odds Ratio
HDL-C level	0.056	1,027
LDL-C level	0.129	0.981
TG level	0.689	1.001
PON1 araoxonase activity	0.027	0.983
PON1 arylesterase activity	0.428	0.992
Diabetes mellitus	0.397	2.597
CC + CT genotype	0.999	0.000
TT genotype	0.521	1.899
Previous myocardial infarct	0.805	1.263
Sex	0.864	0.850
